# Epidemiology and Dynamics of BK Polyomavirus Replication after Kidney Transplantation

**DOI:** 10.3390/pathogens13040315

**Published:** 2024-04-12

**Authors:** Etienne Brochot, Baptiste Demey, Aurélien Aubry, Véronique Descamps, Virginie Morel, Claire Presne, François Brazier, François Helle

**Affiliations:** 1 Department of Virology, Amiens University Medical Center, 80054 Amiens, France; baptiste.demey@u-picardie.fr (B.D.); aubry.aurelien@chu-amiens.fr (A.A.); 2 Agents Infectieux Résistance et Chimiothérapie Research Unit, UR4294, Jules Verne University of Picardie, 80054 Amiens, France; descamps.veronique@chu-amiens.fr (V.D.); morel.virginie@chu-amiens.fr (V.M.); francois.helle@u-picardie.fr (F.H.); 3 Department of Nephrology, Dialysis and Transplantation, Amiens University Medical Center, 80054 Amiens, France; presne.claire@chu-amiens.fr (C.P.); brazier.francois@chu-amiens.fr (F.B.)

**Keywords:** BKPyV, epidemiology, time course, kidney transplantation

## Abstract

Background/Objectives: In the absence of an effective antiviral treatment for BK polyomavirus (BKPyV), a better understanding of the epidemiology and time course of BKPyV replication after kidney transplantation is needed to limit the virus’s impact on the graft outcome. Methods: In a 7-year study, we screened more than 430 kidney transplant recipients and analyzed the time course and virological characteristics of BKPyV replication. Results: Urinary viral replication was observed in 116 (27%) of the 430 patients, and 90 of the 116 (78%) had viral DNAemia. Thirty-eight patients (8.8%) were presumed to have nephropathy (DNAemia > 4 log10 copies/mL). Of the patients with BKPyV replication, 48%, 60%, 71%, and 80% were first found to be positive one, two, three, and four months post-transplantation. The initial viral load in the urine was below 7 log10 copies/mL in 100% of the patients with viral replication first detected before the first month, and this proportion was 57% when viral replication was first detected after the first month. When the BKPyV replication was first detected in a urine sample at month 3 or later, 81.5% of patients had concomitant BKPyV DNAemia. The predominant viral subtype was Ib2 (60%), and there was no apparent relationship between the subtype and the time course of BKPyV replication. Conclusions: Urinary BKPyV replication occurs early after renal transplantation and in most patients will increase to a level requiring therapeutic intervention. Close monitoring for BKPyV in the early post-transplantation period would enable the pre-emptive adjustment of the immunosuppression regimen.

## 1. Introduction

Replication of BK polyomavirus (BKPyV) is a major problem after kidney transplantation. It may lead to nephropathy and thus compromise the graft’s function. The vast majority of nephropathy cases appear within 12 months of transplantation. More recently, greater awareness among clinicians and the availability of more easily accessible, high-performance molecular biology tools have reduced the proportion of BKPyV nephropathy below 10% [1]. Viral replication is initially detected in the urine; if intense replication persists (alteration of the tubular cell leads to the passage of viral DNA into the peri-tubular capillaries), the virus then becomes detectable in the blood (defined as DNAemia). The only treatment option for BKPyV nephropathy is to reduce the immunosuppressive regimen. The clinical practice guidelines issued by the Kidney Disease Improving Global Outcomes (KDIGO) initiative suggest lowering the intensity of the immunosuppressive regimen when the plasma BKPyV load remains above 10^4^ copies/mL [2]. In order to consider this therapeutic option and limit the progression of viral replication toward an irreversible stage, a precise diagnosis is required. The diagnosing physician must be familiar with the advantages and disadvantages of the various techniques used [3]. The strategy used to screen for BKPyV replication depends on the country and the transplant center. Some centers prefer to screen for BKPyV replication in urine samples; others prefer plasma samples, and yet others prefer both matrices. There is a belief in the scientific community that the isolated detection of BKPyV viruria has very few consequences for the patient; this is why some centers do not screen urine samples for viral replication. However, a major disadvantage of the latter strategy may be the loss of an opportunity to identify viral replication at an early stage; in contrast, a pre-emptive diagnostic strategy might enable earlier intervention [4]. Urine testing has a 100% negative predictive value for nephropathy but a lower positive predictive value compared to blood testing. In the KDIGO’s guidelines on pre-emptive screening, tests should be carried out monthly for the first 3–6 months post-transplantation and then every 3 months for the first year [5]. Testing should be repeated more frequently after an unexplained increase in the serum creatinine level or after treatment for acute rejection. Few (if any) large-scale studies have investigated the dynamics of BKPyV replication from the first day post-transplantation to the end of the first year, and, in particular, no data are available from urine samples. The objective of the present study was therefore to evaluate the incidence, dynamics, and viral epidemiology of BKPyV replication in the first year after kidney transplantation in urine and plasma samples. We also sought to evaluate various diagnostic strategies and thus enable clinicians to make rational therapeutic decisions and to better understand the kinetics of BKPyV replication after kidney transplantation.

## 2. Materials and Methods

### 2.1. Study Population and Specimens

We enrolled all consecutive adults having received a kidney transplant between 1 January 2015 and 30 September 2021 at Amiens University Medical Center (Amiens, France) and who had at least one year of follow-up data (Figure S1). The main exclusion criteria were age under 18 and the lack of follow-up data. Our transplantation center screened the patients’ urine samples for BKPyV at post-transplantation day (D)0 and D15 and month (M)1, M2, M3, M4, M6, M9, and M12. If one of the patient’s urine samples tested positive for BKPyV, plasma samples were then tested. A patient was categorized as having replicating BKPyV when two consecutive urine PCR tests were positive. In our medical center, immunosuppressive treatment was adjusted in the case of detection of BKPyV DNAemia.

### 2.2. Clinical and Laboratory Test Data

The clinical, demographic, and laboratory test data for the 430 patients were extracted from the hospital’s electronic medical records. Creatinine clearance data throughout the first year post-transplant were collected as often as available.

### 2.3. BKPyV Subtypes and Subgroups

Viral subtypes and subgroups were determined using a method developed and described in our laboratory. The method is based on a viral genomic fragment called the BKPyV virus typing and grouping region (BKTGR) [6].

### 2.4. Quantification of the BKPyV Load

Nucleic acids were extracted from 200 µL of urine or plasma and amplified in a quantitative real-time PCR assay (the R-gene kit from Argene, Verniolle, France, or the RealStar BKV PCR kit 1.0 from Altona Diagnostics, Cabildo, France, according to the protocols described [7]).

### 2.5. Data Analysis and Statistical Analyses

Frequency of outcomes was used for qualitative parameters. Categorical data are expressed as number (%). Median and interquartile ranges were used for quantitative variables. Frequencies of outcomes in the group of cases were compared using Fisher’s exact test, or with chi-squared test if multiple exposures were simultaneously analyzed. Because of skewed distribution of variables, quantitative variables were compared using non-parametric Mann–Whitney test. To compare 3 or more groups in the same time, Kruskal–Wallis test was performed. All statistical analyses were performed with GraphPad Prism software (version 5, GraphPad Software LLC, San Diego, CA, USA). The threshold for statistical significance was set to *p* < 0.05.

## 3. Results

### 3.1. Epidemiology of BKPyV in the First 12 Months Post-Transplantation

**Figure 1:**
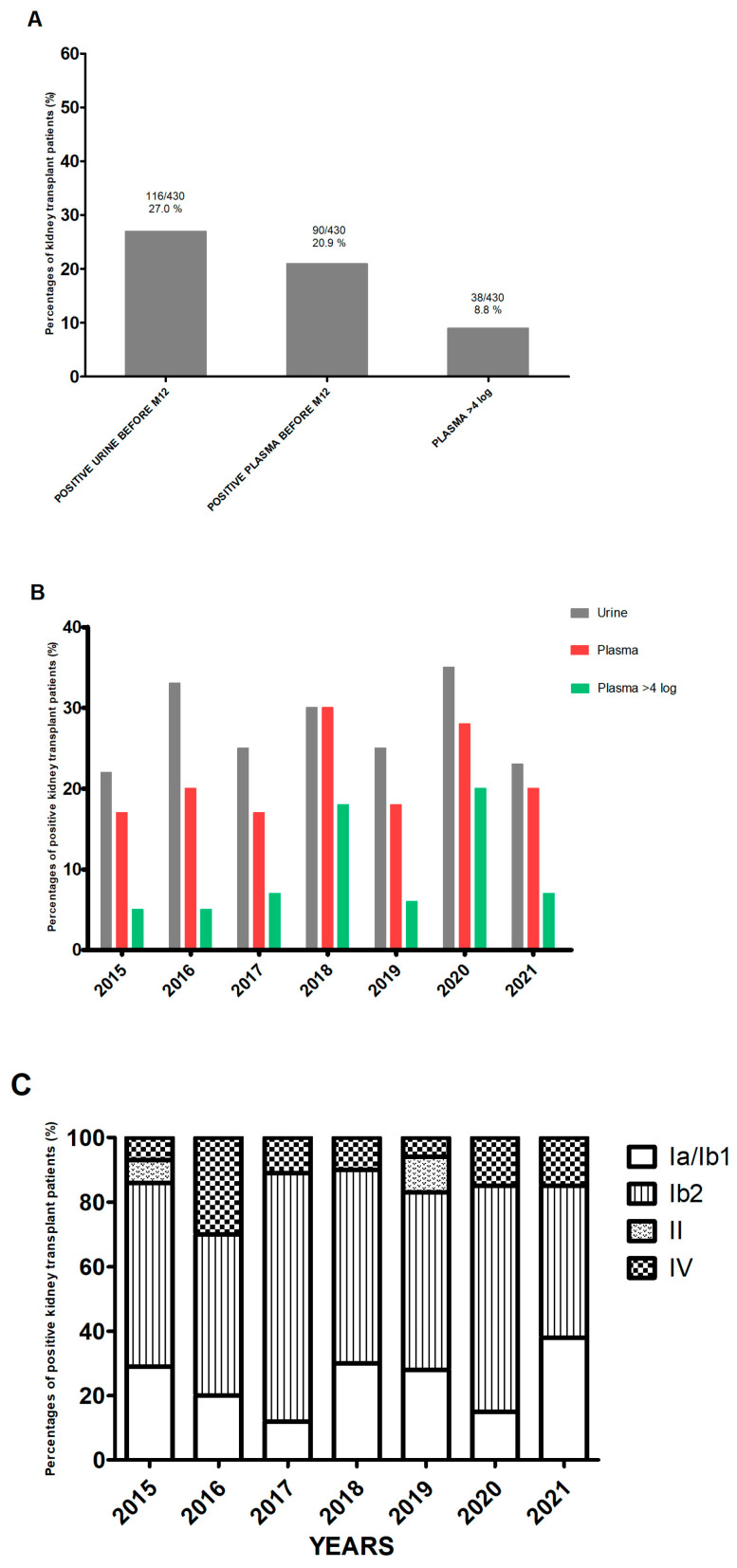
Epidemiology of BKPyV. (**A**) The proportion of patients with BKPyV replication in a PCR test, by sample type. (**B**) The frequency of BKPyV detection, by year of transplantation. (**C**) The frequency of viral subtypes, by year of transplantation.

During the 7-year study period (from 1 January 2015 to 30 September 2021), 430 patients from our center were tested routinely in the first 12 months after transplantation (Figure S1). Of the 430 patients, 116 (27.0%) had PCR-positive urine samples. Ninety patients (77.6% of the 116 with positive urine samples and 20.9% of the total study cohort) also had PCR-positive plasma samples, i.e., DNAemia (Figure 1A). Thirty-eight patients (8.8%) were presumed to have nephropathy because the DNAemia exceeded 4 log10 copies/mL [8]. The characteristics of patients in the three defined groups (urine- and plasma-negative (U−/P−), urine-positive/plasma-negative (U+/P−), and urine- and plasma-positive (U+/P+)) are summarized in Table 1. There were no significant differences between the three groups, apart from the recipient’s age and the donor’s age, which were higher in the U+/P+ group. Over the study period, by year of transplant, the proportion of patients with a positive urine test was above 20% each year and above 30% in 2016 (32.8%) and 2020 (35%) (Figure 1B). The proportion of patients with a positive plasma test was above 17% in all seven years of grafting and peaked in 2020 (27.5%) and 2018 (29.9%). In 2018, all transplant patients with a positive urine test also had a positive plasma test. The proportion of patients with a viral load above 4 log10 copies/mL also varied markedly from one year to another and ranged from 4.9% in 2016 to 20% in 2020. Lastly, with regard to the viral subtypes found over these 7 years, it came as no surprise that subtype Ib2 accounted for between 38% (in 2021) and 76.5% (in 2017) of the strains. Subtypes II and IV accounted for 2.6% and 13.8% of the strains, respectively (Figure 1C).

### 3.2. The Time Course of BKPyV Replication after Kidney Transplantation

**Figure 2:**
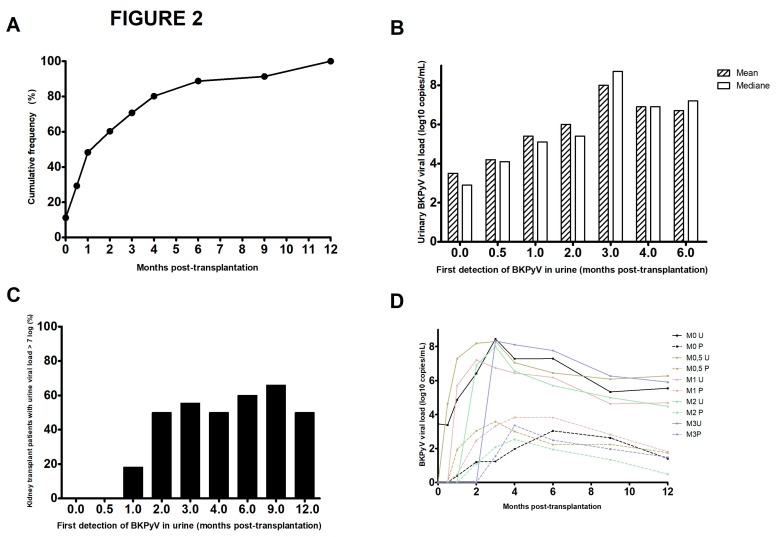
The time course of BKPyV replication over the first two years post-transplantation. (**A**) Cumulative frequency of urine-based detection of BKPyV replication among the 116 patients screened, as a function of the time interval since kidney transplantation. (**B**) Mean and median urine viral loads at first detection of viral replication. (**C**) Percentages of BKPyV-positive patients with a urine viral load >7 log10 copies/mL, as a function of the time of first virus detection. (**D**) Mean urine and plasma viral loads, according to the time of first detection of viral replication.

As mentioned in the Materials and Methods section, the patients in our center are tested repeatedly in the first year post-transplantation. This strategy gave us a better view of the status of 116 patients showing viral replication in urine samples during this period. We found that the sample taken a few hours after transplantation was positive in 8.6% of the 116 patients (Figure 2A) and that 48.4% of the 116 patients were positive at M1 post-transplantation. The proportion rose to 60.3% (*n* = 70 out of 116), 70.7% (*n* = 82), and 80.2% (*n* = 93) at M2, M3, and M4, respectively. The median or mean urine viral load (depending on the time of first detection) rose over time to around 8 log10 copies/mL when replication was first detected at M3 (Figure 2B). When viral replication was first detected at D0 or D15, none of the patients had a viral load above 7 log10 copies/mL (Figure 2C). The proportion of patients with this viral load threshold was 18.2% (*n* = 4 out of 22) when replication was first detected at M1 and around 50% when replication was first detected at other time points. With regard to the frequency of onset of DNAemia as a function of the viral subtype, we did not observe any significant difference. However, two nonsignificant trends emerged: a lower frequency of DNAemia in cases of subtype II replication and a higher frequency of DNAemia above 4 log10 copies/mL for the genotype IV (Figure S2).

By analyzing the changes over time in urine and plasma viral loads for the 116 patients with BKPyV replication within 12 months of transplantation, we observed that the increase in viral load was very rapid after a first urine detection at D15, M1, M2, or M3 but was much slower after the first urine detection a few hours after transplantation and takes 3 months to reach peak viral load (Figure 2D). The same trend was observed for the time course of the plasma viral load. Thus, early detection of viral replication is not predictive of more intense replication.

### 3.3. Impact of BKPyV Replication on Renal Function

**Figure 3:**
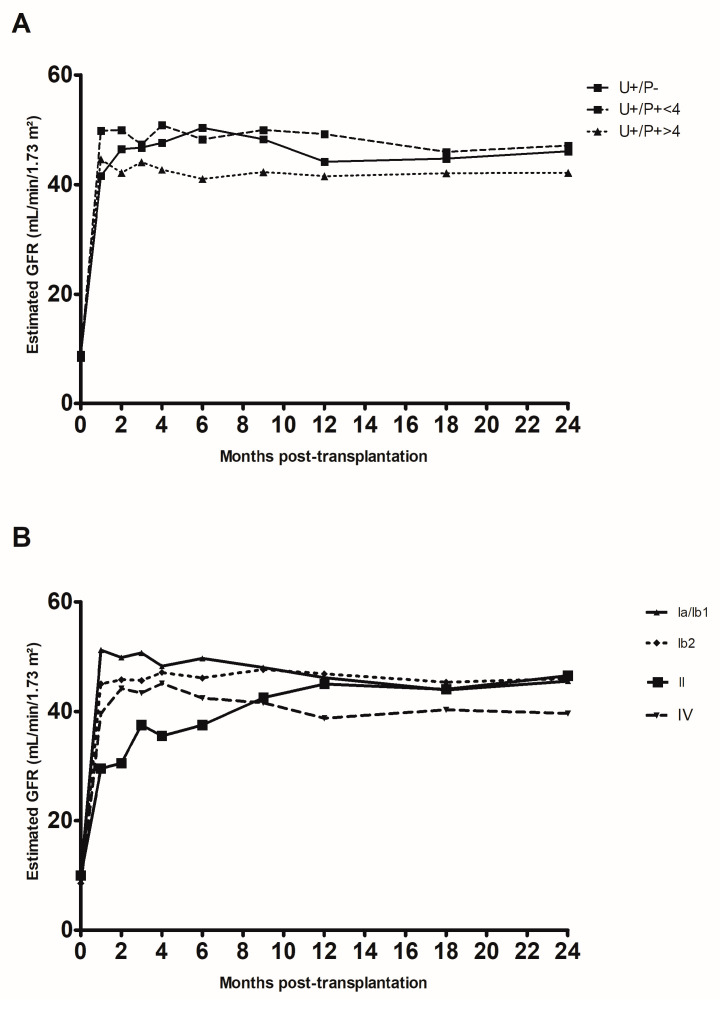
Change over time in creatinine clearance rate (estimated according to the Modification of Diet in Renal Disease formula) in the first two years after kidney transplantation. (**A**) Changes according to the patient group: positive in urine only (U+/P−), positive in urine and plasma with a viral load below 4 log10 copies/mL (U+/P− < 4, and positive in urine and plasma with a viral load above 4 log10 copies/mL (U+/P− > 4). (**B**) Changes according to the viral subtypes detected.

Lastly, we assessed the impact of replication on renal function up to two years after transplantation in three subgroups: the U+/P− group, the members of the U+/P+ group with a plasma load below 4 log10 copies/mL, and the members of the U+/P+ group with a plasma load above 4 log10 copies/mL (Figure 3A). Although we did not observe any significant difference between the three subgroups, we noted that the estimated glomerular filtration rate (eGFR) was lower in the U+/P+ > 4 log10 copies/mL subgroup than in the two other subgroups. When considering the viral subtype (Figure 3B), only the subtype IV showed a lower eGFR (up to 24 months post-transplantation) than the other subtypes. This was probably related to the higher plasma viral load found for this subtype (Figure S2).

## 4. Discussion

Our study on over 400 kidney transplant recipients provided robust, precise data on the onset of BKPyV replication. Our results demonstrate that in a large proportion of patients, viral replication starts very soon after transplantation (Figure 2). Of the patients who were positive for BKPyV at the end of the first year post-transplantation, 50% had an onset by M1, and 80% had an onset by M4. In more than 77% of patients with a positive urine sample, the viral genome was subsequently found in the plasma. The trigger for BKPyV replication has not yet been characterized, although a state of immunosuppression is necessary. Some researchers have suggested that factors including the use of corticoids, the use of stents, the cold ischemia time, and older recipient age favor BKPyV replication [9,10]. BKPyV has a broad cell infection tropism but optimized replication in dividing cells only, and this phenomenon is particularly intensified after cellular aggression [11].

We looked at whether the virological data varied from one year to another (Figure 1) and found that the percentage of patients with BKPyV replication was always greater than 20%. In one year only (2018), all patients with a positive urine test also had a positive plasma test; we do not have an explanation for this phenomenon. The year 2020 marked the start of the coronavirus 2019 pandemic; relative to other years, there were fewer kidney transplantations, a higher prevalence of BKPyV (close to 40%), and a high proportion of patients with presumptive nephropathy (DNAemia > 4 log10 copies/mL). The disruption of the healthcare system and thus a decrease in the extent and frequency of post-transplantation follow-up surely contributed (at least in part) to this situation. Furthermore, we observed that late first detection of replication was associated with a higher proportion of patients with a viral load above 7 log10 copies/mL (Figure 2C); this was probably related to longer time intervals between consultations, which gave the virus more time to replicate undetected. None of the 116 patients with BKPyV replication managed to decrease their viral load in the absence of therapeutic intervention. Once replication had started, it intensified rapidly or stabilized at a fairly high level. The epidemiological data on patients with BKPyV replication are perfectly aligned with the literature from European countries [12]. A point that would probably warrant the implementation of a large multicenter study concerns subtype IV, which accounts for barely 15% of the BKPyV-positive patients but appeared to be more frequently present when the plasma viral load exceeded 4 log10 copies/mL (Supplementary Figure S2). The median peak was 4.1 log10 copies/mL for subtype IV versus 3.5 log10 copies/mL for subtypes Ia/Ib1 and 3.7 log10 copies/mL for subtype Ib2. Multicenter studies on genotype IV would be interesting, because data in the literature point to a greater pathogenicity of this subtype [13,14].

The strategy for the screening and/or diagnosis of BKPyV replication after kidney transplantation differs markedly from one country to another, one transplantation center to another, and even among clinicians at the same center. For example, a study of a pediatric population of kidney transplant recipients in Europe highlighted urine-only screening in 26% of patients, urine and blood screenings in 37%, and blood-only screening in 37% [15]. Our data show that the detection of viral replication in the urine before M1 (Figure 2C) was a warning sign for the clinician and gave him/her more time to implement a change in the immunosuppression regimen. Recent data indicate that clearance of BKPyV after a reduction in immunosuppression is easier when the DNAemia is below 4 log10 copy/mL [16]. Other studies have shown that early, frequent detection of BKPyV replication prompts rapid intervention and is associated with better transplantation outcomes [17]. In the future, it would be interesting to consider a randomized study of immunosuppressive therapy modification early after detection of BKPyV urinary replication, or later after detection of BKPyV DNAemia, and to understand the risk of nephropathy and the rejection or appearance of donor-specific antibodies. Some researchers have suggested that a delayed T cell response has a detrimental role [18]. Indeed, T cells accumulate in kidney transplants with BKPyV nephropathy and speed the progression to graft loss [19]. The uncontrolled replication of BKPyV is thought to cause chronic inflammation, which in turn leads to an influx of activated T cells that attack the graft’s cells (whether infected or not) and cause extensive lesions. Surprisingly, 13 patients presented with DNAuria a few hours after the transplant (Figure 2). All 13 subsequently presented a slow increase in the urine viral load (Figure 2D); this suggests the presence of a state of equilibrium between the virus and the immune system and might give the physician an opportunity to act before DNAemia appears.

Our study had some limitations. Firstly, it was a retrospective analysis of a mainly Caucasian population. Secondly, we only studied viral replication the first year post-transplantation, whereas late replications have been reported in the literature. In our cohort, six patients (1.4%) began intense BKPyV replication after the first year post-transplantation.

## 5. Conclusions

Our results emphasize the importance of frequent screening for BKPyV replication in the first few months post-transplantation. It must be borne in mind that sooner or later, nearly 80% of patients with DNAuria will have DNAemia requiring a therapeutic intervention. Thus, in the current absence of an effective antiviral therapy, good knowledge of the intensity and time course of BKPyV replication will help to improve the outcome of kidney transplantation.

## Figures and Tables

**Table 1:** Characteristics of the study population.

	BKPyV-Negative (U−/P−) *n* = 314	BKPyV Urine-Positive (U+/P−) *n* = 26	BKPyV Urine- and Plasma-Positive (U+/P+) *n* = 90	*p*-Value
Recipient age (Median (IQR))	53 (14.0)	45.2 (13.7)	53.7 (12.5)	NS ^1^
				0.02 ^2^
				0.002 ^3^
Recipient sex (M/F) (%/%)	214/110 (68.2/31.9)	18/8 (69.2/30.8)	60/30 (66.6/33.3)	NS
Living donor (*n*) (%)	32 (10.2)	2 (7.7)	6 (6.66)	NS
Donor age (Median (IQR))	52.7 (2.0)	49.5 (16.6)	56.4 (13.0)	0.02 ^3^
Donor sex (M/F) (%)	177/137 (56.4/43.6)	18/8 (69.2/30.8)	51/39 (56.6/43.4)	NS
Recipient blood type (*n*) (%)				NS
A	151 (48.0)	11 (42.3)	30 (33.3)	
B	32 (10.2)	2 (7.7)	8 (8.9)	
O	124 (39.5)	11 (42.3)	49 (54.5)	
AB	7 (2.3)	2 (7.7)	3 (3.3)	
Body mass index (Median (IQR))	26.3 (4.2)	25.7 (3.7)	26.0 (4.4)	NS
HLA mismatch (median)	6	6	6	NS
Induction (*n*, (%))				NS
Thymoglobulin	117 (37.3)	10 (38.5)	35 (38.8)	
Basiliximab	197 (62.7)	16 (61.5)	55 (61.2)	
Maintenance treatment (*n*, (%))				NS
Cyclosporine	51 (16.2)	3 (11.5)	11 (12.2)	
Tacrolimus	262 (83.4)	23 (88.5)	79 (87.7)	
Corticoids	193 (61.5)	15 (57.7)	47 (52.2)	
BKPyV subtype subgroups (*n*, (%))	NA			NS
Ia/Ib1		8 (30.7)	20 (22.2)	
Ib2		14 (53.9)	55 (61.1)	
II		2 (7.7)	1 (1.1)	
IV		2 (7.7)	14 (15.6)	

NA: not applicable. NS: not significant. ^1^ U−/P− vs. U+/P−. ^2^ U−/P− vs. U+/P+. ^3^ U+/P− vs. U+/P+.

## Data Availability

The data presented in this study are available on request from the corresponding author.

## Supplementary Materials

The following supporting information can be downloaded at https://www.mdpi.com/article/10.3390/pathogens13040315/s1, Figure S1: Flow chart of the patient cohort over the 7-year study period; Figure S2: Proportion of kidney transplant recipients (%) with a BKPyV DNA load above or below 4 log10 copies/mL, by viral subtype.
