# Supplementary figures and images for: Epidemiology and Dynamics of BK Polyomavirus Replication after Kidney Transplantation
Source: Pathogens. 2024 Apr 12;13(4):315. doi: 10.3390/pathogens13040315 (PMC11053930; doi:10.3390/pathogens13040315)

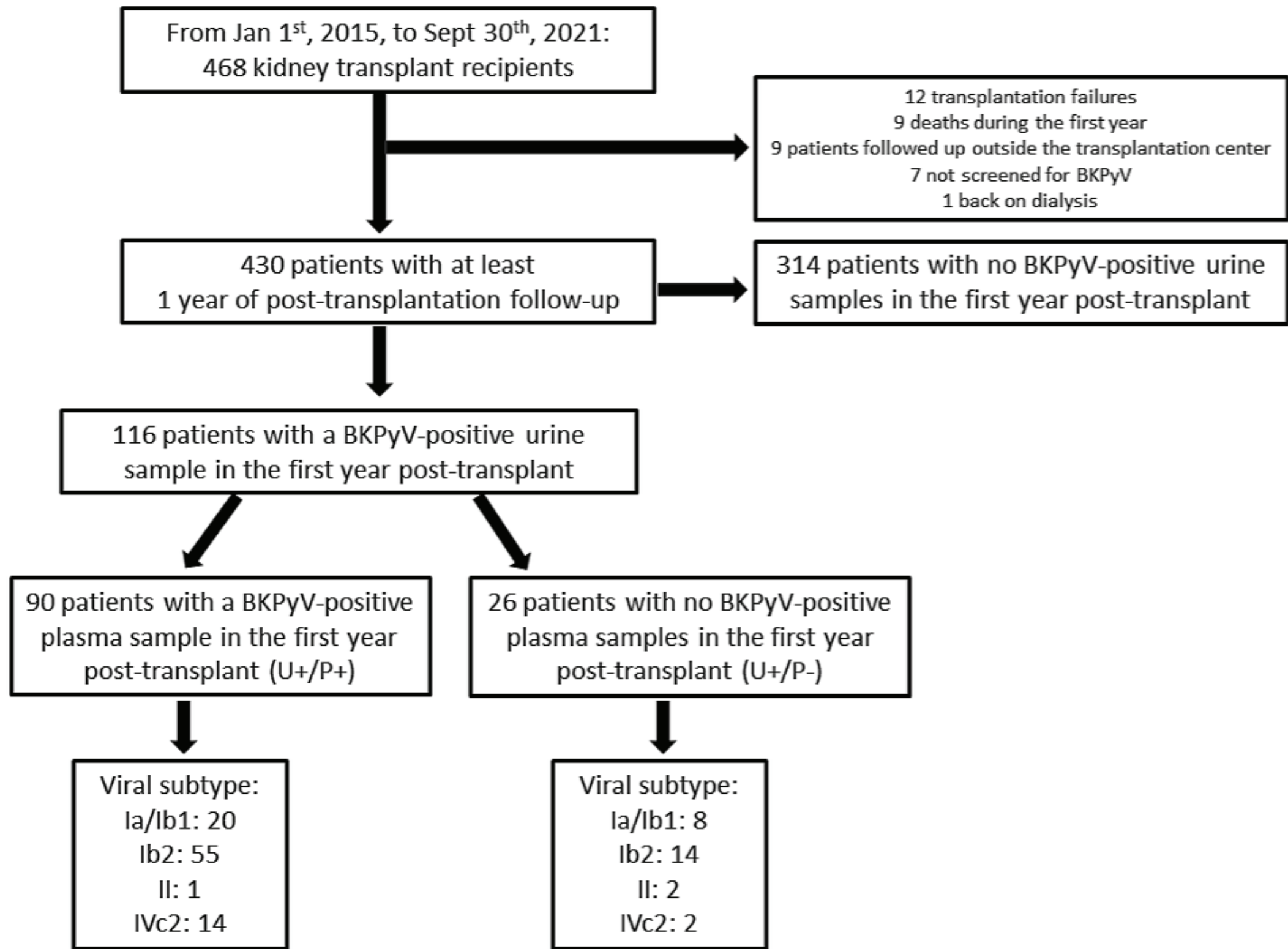

Supplement: Supplementary file 1 [file pathogens-13-00315-s001.zip › Figure S1.pdf]

FIG S2

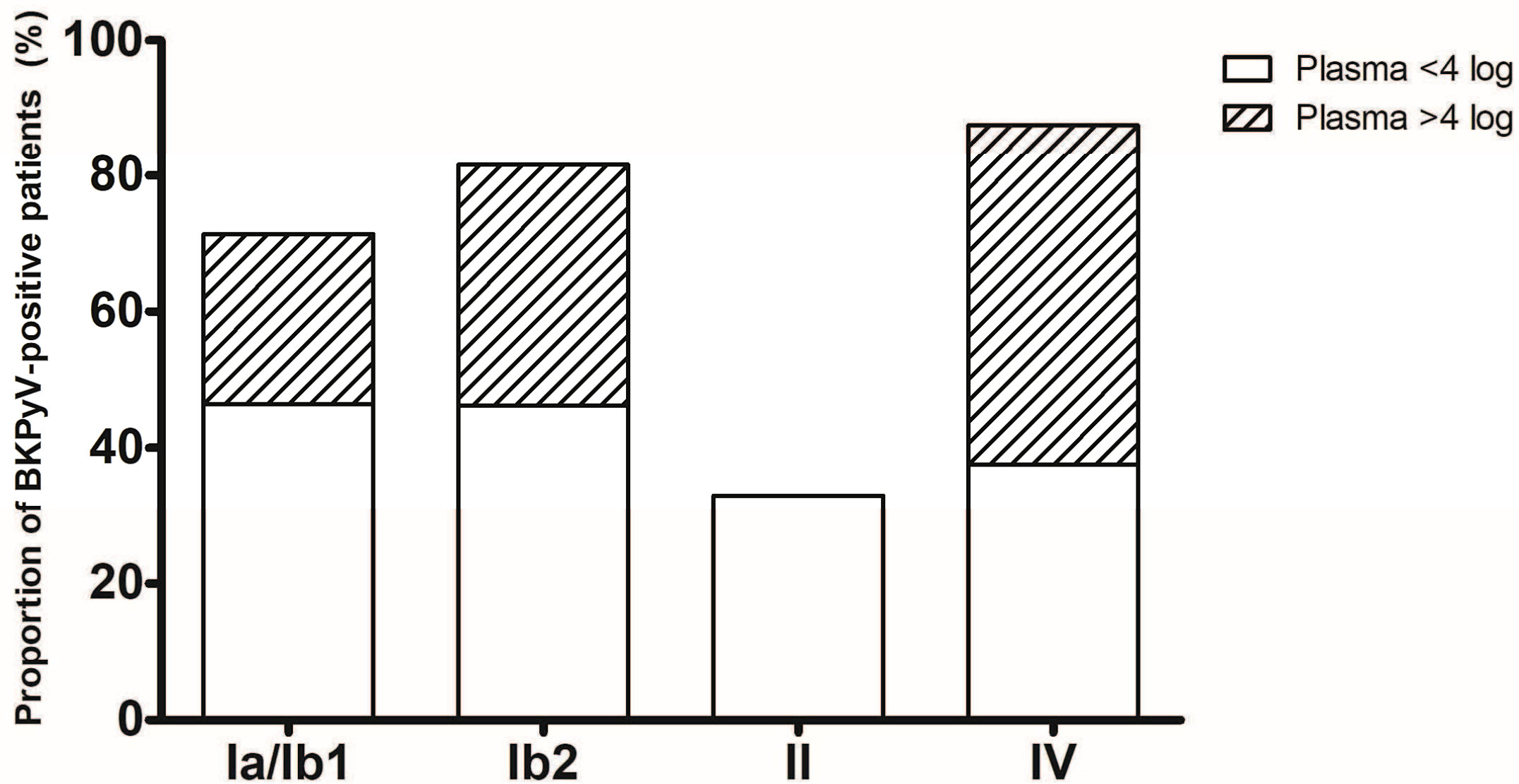

Supplement: Supplementary file 1 [file pathogens-13-00315-s001.zip › Figure S2.pdf]
